# Estrogen receptor positivity in stromal cells of the tumor bed predicts the response to neoadjuvant chemotherapy for breast carcinoma

**DOI:** 10.1007/s10549-024-07601-6

**Published:** 2025-01-08

**Authors:** Aysel Bayram, Sidar Bagbudar, Hasan Karanlık, Neslihan Cabıoglu, Adnan Aydıner, Semen Onder, Ekrem Yavuz

**Affiliations:** 1 Department of Pathology, Istanbul Faculty of Medicine, Istanbul University, 34390 Faith, Istanbul, Turkey; 2 Department of Surgical Oncology Unit, Institute of Oncology, Istanbul University, Istanbul, Turkey; 3 Department of General Surgery, Istanbul Faculty of Medicine, Istanbul University, Istanbul, Turkey; 4 Department of Medical Oncology, Institute of Oncology, Istanbul University, Istanbul, Turkey

**Keywords:** Neoadjuvant chemotherapy, Breast carcinoma, Tumor bed, Stromal cell, Estrogen receptor

## Abstract

**Purpose:**

This study aimed to determine estrogen receptor (ER) expression in stromal cells in postchemotherapy tumor bed (PCTB) and its relationship with tumor regression and tumor characteristics.

**Methods:**

The study included 490 breast cancer patients who received neoadjuvant chemotherapy (NAC). We performed ER in stromal cells in all resection specimens and available pre-treatment core biopsy materials of 299 patients immunohistochemically.

**Results:**

Two hundred and forty-two (49.4%) cases were negative for ER in the stromal cells of the PCTB, and 248 (50.6%) cases were positive. ER-positive stromal cells in the PCTB correlated with a higher regression rate (90.2 vs 68.6%) and lower mean residual cancer burden value (1.366 vs 2.424) compared to ER-negative cases (*p* < 0.001). Stromal ER positivity was more prevalent in cases achieving pathologic complete response (pCR) (68.1%) compared to those without pCR (39.8%, *p* < 0.001). ER positivity in stromal cells was more common in non-luminal tumors than in luminal ones. Multivariate analysis identified stromal ER positivity (OR: 3.059, 95% CI [1.947–4.807], *p* < 0.001), intrinsic subtype (Odds ratio (OR): 1.477, 95% confidence interval (CI) [1.102–1.980], *p* = 0.009), and Ki67 index (OR: 1.028, 95% CI [1.104–1.041], *p* < 0.001) as independent predictors of pCR. In core biopsies before NAC, 270 cases (90.3%) and 29 cases (9.7%) were negative and positive in stromal cells, respectively. Out of the cases with ER-negativity in stromal cells before NAC, 132 (48.9%) converted to ER positivity in stromal cells of PCTB and displayed a high regression rate (89.8%).

**Conclusion:**

This is the first study regarding ER expression in the stroma of breast carcinoma that compares treatment response after NAC. We showed that the increase in ER positivity in the stromal cells of the PCTB is correlative with the complete response and tumor subtypes. In this manner, ER positivity in stromal cells will soon serve as a cornerstone for individualized treatment options.

**Supplementary Information:**

The online version contains supplementary material available at 10.1007/s10549-024-07601-6.

## Introduction

Neoadjuvant chemotherapy (NAC) prior to surgery has become the standard of treatment in breast carcinoma cases with advanced stage and negative biologic characteristics, giving way to the opportunity to assess and quantify the response in the subsequent resection specimen. There are several models for tumor regression scoring, of which some take into account the amount of reactive stroma within the tumor bed [1–6]] However, so far, the details of stromal components after NAC for breast carcinoma have not been well-documented.

Microscopically, the postchemotherapy tumor bed (PCTB) in breast carcinoma is characterized by an irregular area of vascularized fibrous stroma that either lacks or has scant normal ducts and lobules. It may exhibit varying degrees of edema, myxoid change, fibroblastic/myofibroblastic proliferation, elastosis, residual calcification, and inflammatory cells [7]. Before and after NAC, the tumor stroma mainly comprises cancer-associated fibroblasts (CAFs), characterized as large, spindle-shaped mesenchymal cells [8]. Myofibroblasts are another type of stromal cells activated during the wound healing and fibrosis processes in inflammation [9]. CAFs are believed to be the activated type of myofibroblasts in tumors, expressing alpha-smooth muscle actin (α-SMA) [10, 11]. Despite extensive research, a specific marker for CAFs has yet to be identified. Recent studies concerning CAFs have proven their significant role in cancer invasion and metastasis by directly stimulating cancer cell proliferation through the secretion of various growth factors and cytokines [12–14].

For several years, during the examination of the breast specimens resected after NAC, we observed an unexpected estrogen receptor (ER) expression in the stromal cells (probably CAFs) among the residual carcinoma cells in the PCTB. When we checked the literature, we did not find any study regarding ER expression in the stromal cells of breast carcinoma. Hence, this study was designed to scrutinize the relationship of ER expression in the stromal cells in breast carcinoma after NAC.

## Material and methods

### Case selection

Of the 3437 invasive breast carcinoma cases diagnosed in the Department of Pathology at Istanbul Medical Faculty between 2018 and 2021, 505 cases that received NAC were retrieved from the Pathology department’s computer archive. Of these, 490 cases with sufficient tissue for immunohistochemical analysis were included in this study. Clinical features such as age, menopausal status, clinical stage, and neoadjuvant chemotherapy protocols were gathered from the patient’s charts at relevant clinics.

The surgically removed breast tissue was sent fresh for pathological examination, thinly sliced, and promptly fixed in buffered formalin. If the tumor was detectable macroscopically, its size was measured. If there was a large (> 3 cm) apparent PCTB on gross examination, at least one sample per centimeter of carcinoma size was taken. If the remaining PCTB was small (< 3 cm), the entire fibrous area was sampled for pathological examination. If no gross disease was found, widespread sampling of the involved quadrant was undertaken and guided by the tumor's pre-operative radiological location. In the microscopic examination, the area in the tumor bed where the tumor disappeared and regressional changes remained was called 'stromal fibrosis compatible with regression'. Thus, the regression rate was determined by evaluating all slides for each case. We also used the published method to evaluate the residual cancer burden (RCB) and the associated website to calculate RCB [6] (www.mdanderson.org/breastcancer_RCB).

In 470 cases, ER, progesterone receptor (PR), Human Epidermal Growth Factor Receptor 2 (HER2), and Ki67 examination results were obtained from the patient's pathology reports, which were studied immunohistochemically in pre-treatment core biopsy samples. Accordingly, the cases were grouped as luminal A-like (ER + /PR + /HER2 − /Ki67 low), luminal B-like (ER + /PR + /HER2 − or + / Ki67 high), HER2 overexpression (ER − /PR − /HER2 +), and triple-negative (ER − /PR − /HER2 −).

Pre-treatment core biopsy samples were also re-examined in our department from 299 out of these 490 patients. ER reactivity in the tumors' stromal cells was evaluated, and any nuclear staining in stromal cells was recorded as positive.

### Immunohistochemical analysis

Whole tissue blocks were used in the study. A proper paraffin block containing the tumor bed was identified for each case, and immunostaining was performed on 4 µm paraffin-embedded tumor tissue sections. After deparaffinization, monoclonal antibody against the ER (clone SP1, 1:100 dilution; Biocare Concord, CA, USA) was applied to tissue sections. Two experienced pathologists (EY, AB) evaluated ER-stained slides. Immunostaining in the nucleus was regarded as ER positivity. The proportion of ER-positive stromal cells in the tumor bed was recorded.

### Statistical analysis

The receiver operating characteristics (ROC) curve and the calculation of the area under the curve (AUC) for ER staining were performed. The ROC curves were plotted as 1 minus specificity versus sensitivity for patients who had pathologic complete response (pCR) and not pCR classified as RCB to obtain precise sensitivity and specificity at various cut-off levels of ER. The ROC curve derived a cut-off of 55% with 68.1 percent sensitivity and 60.2 percent specificity (AUC 0.647 95% CI 0.590–0.703 *P* < 0.001. ER staining was categorized as positive or negative according to the 55% cut-off value (Supplement).

Categorical variables were analyzed using the Chi-square test or Fisher exact test where appropriate. Odds ratio (OR) with 95% confidence interval (CI) for categorical variables were also determined. Continuous variables are given as mean (± SD) and were analyzed using the Mann–Whitney *U* test. Subsequently factors associated with tumor regression were included in multivariate stepwise logistic regression analysis.

All statistical analyses were performed using Statistical Package for Social Sciences (SPSS) software for Windows, version 21.0 (IBM Corp, Armonk, NY, USA). *p* value less than 0.05 was considered significant in all comparisons.

## Results

The mean age of 490 patients with breast cancer treated with NAC was 48.6 (range: 25–82). Of the patients, 262 (53.5%) were pre-menopausal and 228 (46.5%) were post-menopausal. The initial T stages of the cases (T1, T2, T3, T4) were 68 (13.9%), 269 (54.9%), 115 (23.5%), and 38 (7.7%), respectively. The lymph node was tumor-free in 259 (52.8%) cases and metastatic in 231 (47.2%). Before NAC, the intrinsic subtype was determined according to ER, PR, HER2 status, and Ki67 indices in 470 cases. Among them, 53 (11.3%) cases were luminal A-like subtype, 212 (45.1%) cases were luminal B-like subtype, 123 (26.2%) cases were HER2 overexpression subtype, and 82 (17.4%) cases were triple-negative subtype. The mean regression rate of 490 patients with breast cancer treated with NAC was 79% (range: 10–100). Two hundred (40.8%) cases had pathologic complete response (pCR) in the PCTB in breast. There were 166 (33.9%) cases with RCB class 0 (pCR), 24 (4.9%) cases with RCB class 1, 151 (30.8%) cases with RCB class 2, and 149 (30.4%) cases with RCB class 3.

**Fig. 1:**
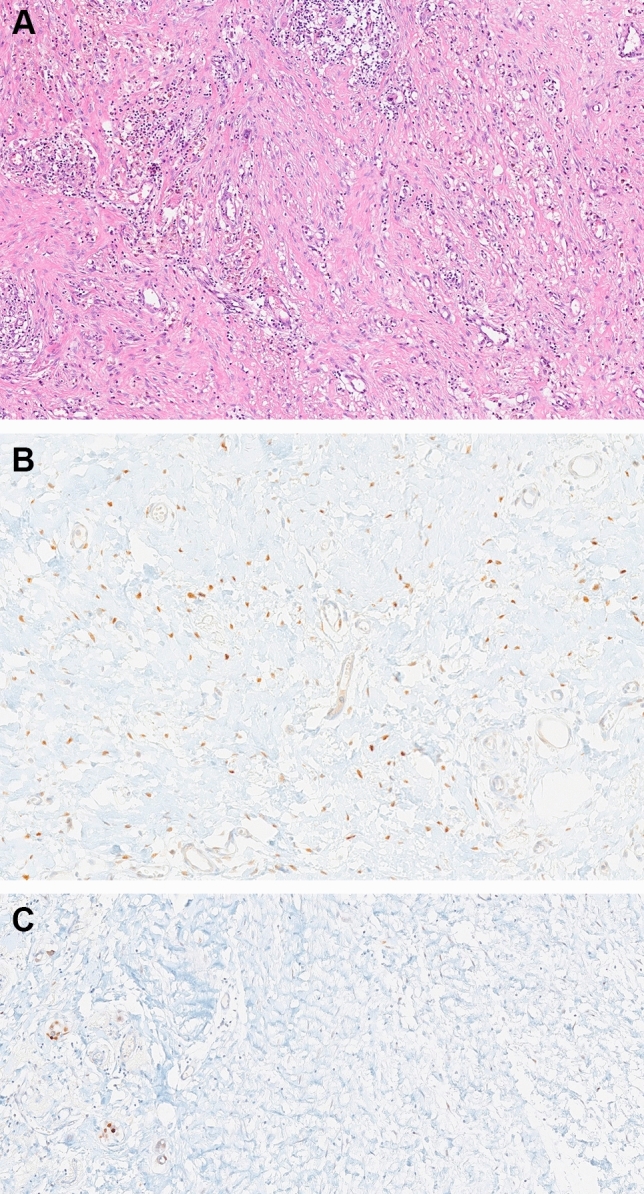
**a** The tumor bed is an irregular area of vascularized fibrous stroma that exhibits varying degrees of fibroblastic/myofibroblastic proliferation and inflammatory cells and is devoid of normal ducts and lobules. (× 200). **b** Estrogen receptor positivity in the stromal cells of the tumor bed (× 400). **c** Estrogen receptor negativity in the stromal cells of the tumor bed (× 400)

Of the postchemotherapy cases, 242 (49.4%) and 248 (50.6%) were negative and positive, respectively, for ER in the stromal cells of PCTB (Fig. 1). The mean regression rate was 68.6 and 90% for cases with ER-negative and ER-positive stromal cells in the PCTB, respectively, and the difference was statistically significant (*p* < 0.001). Of the 200 cases with pCR, 60 (30%) were negative, and 140 (70%) showed positivity for ER in stromal cells of the PCTB, respectively (*p* < 0.001).

According to the threshold value determined by ROC, the mean RCB value was 1.366 and 2.424 for cases with ER-positive and ER-negative stromal cells in PCTB, respectively (*p* < 0.001). The stromal ER positivity rate was 68.1% in cases with pCR in the RCB system, while the stromal ER positivity rate was 39.8% in those without complete response (*p* < 0.001) (Table 1).

**Table 1:** Distribution of ER staining in stromal cells after neoadjuvant treatment according to clinical and pathological features

	All Patients (*n* = 490) (%)	Stromal ER-negative (*n* = 248) (%)	Stromal ER-positive (*n* = 242) (%)	*p*
Age (mean)	48.6	49.1	48.2	0.383
Clinical tumor diameter (mm) (mean)	39.4	40.1	38.7	0.530
Clinical T stage				0.054
T1	68 (13.9)	43 (63.2)	25 (36.8)	
T2	269 (54.9)	138 (51.3)	131 (48.7)	
T3	115 (23.5)	52 (45.2)	63 (54.8)	
T4	38 (7.7)	15 (39.5)	23 (60.5)	
Subtype				< *0,001*
Luminal A-like	53 (11.3)	43 (81.1)	10 (18.9)	
Luminal B-like	212 (45.1)	110 (51.9)	102 (48.1)	
HER2 overexpression	123 (26.2)	51 (41.5)	72 (58.5)	
Triple-negative	82 (17.4)	35 (42.7)	47 (57.3)	
Ki67 (%) (mean)	38.2	36.1	40.5	*0,019*
Regression rate in tumor bed (%)	79.2	68.6	90	< *0,001*
RCB class				< *0,001*
0 (pCR)	166 (33.9)	53 (31.9)	113 (68.1)	
I	24 (4.9)	6 (25)	18 (75)	
II	151 (30.8)	88 (58.3)	63 (41.7)	
III	149 (30.4)	101 (67.8)	48 (32.2)	
Chemotherapy regimens				*0.021*
Antracycline	20 (6.4)	13 (65)	7 (35)	
Antracycline + taxane	245 (50)	135 (55.1)	110 (44.9)	
Antracycline + taxane + trastuzumab	123 (26.2)	51 (41.5)	72 (58.5)	
Antracycline + taxane + carboplatin	82 (17.4)	35 (42.7)	47 (57.3)	

Significant *p*-values are given in italic face

The average regression rates after NAC were 48.7% in the luminal A-like subtype, 77% in the luminal B-like subtype, 86.2% in the HER2 overexpression subtype, and 88% in the triple negative subtype, respectively (*p* < 0.001). ER expression in the stromal cells of the PCTB was positive in 18.9, 48.1, 58.5, and 57.3% of cases of luminal A-like, luminal B-like, HER2 overexpression, and triple-negative subtypes, respectively (*p* < 0.001). When the cases were classified as luminal and non-luminal subtypes, the difference in ER positivity in the stromal cells of the PCTB was statistically significant between the two groups (42.3 vs 58%) (*p* < 0.001) (Table 1).

According to pre-treatment radiological findings, the mean tumor diameter in cases with pCR according to RCB was 37.7 ± 1.7, while the mean tumor diameter in cases with incomplete response was 40.3 ± 1.4 (*p* = 0.256). In addition, according to RCB, the mean Ki67 value in cases with pCR was 47.9%, while the mean Ki67 value in cases with incomplete response was 33.7% (*p* < 0.001). Significant or near significant factors found by univariate test were included in multivariate stepwise logistic regression analysis. In the multivariate logistic analysis performed by selecting stromal ER positivity, subtype, Ki67 index, age, and tumor diameter as covariates for predicting pCR, stromal ER positivity (OR: 3.059; 95% CI [1.947–4.807]; *p* < 0.001), intrinsic subtype (OR: 1.477; 95% CI [1.102–1.980]; *p* = 0.009), and Ki67 index (OR: 1.028; 95% CI [1.104–1.041]; *p* < 0.001) were found to be independent factors (Table 2).

**Table 2:** Multivariate logistic regression analysis regarding pathologic complete response

	Univariate			Multivariate		
Variables	OR	95%CI	*p*	OR	95% CI	*p*
Age (year)	1.637	0.301–0.684	0.077	1.006	0.986–1.027	0.544
Tumor diameter (millimeter)	1.861	0.540–0.702	0.256	1.003	0.994–1.013	0.496
Subtype (Luminal vs non-luminal)	2.824	1.904–4.188	< *0.001*	1.477	1.102–1.980	*0.009*
Ki67 index (percentage)	2.674	0.895–1.072	< *0.001*	1.028	1.104–1.041	< *0.001*
Stromal ER positivity (positive vs negative)	3.223	2.171–4.784	< *0.001*	3.059	1.947–4.807	< *0.001*

Significant *p*-values are given in italic face

*OR* odds ratio; *CI* confidence interval

ER expression in the tumor stroma was analyzed in core biopsy before the NAC in 299 cases. Among them, 270 (90.3%) and 29 cases (9.7%) showed ER negativity and positivity in stromal cells, respectively. Of the 270 cases with stromal ER negativity before NAC, 132 cases (48.9%) converted to ER positivity in stromal cells of the PCTB after NAC. The regression rate of those that remained negative for ER (138/270) after NAC was 68.4%, while the regression rate of those that changed to positive was 89.8% (*p* < 0.001). Pathologic complete response rates were also found to be 23.2 and 56.8% in the ER negative-remaining and ER-positive/conversion groups, respectively (*p* < 0.001). We also identified five cases in which stromal cells were ER-positive in core biopsy and turned ER-negative after neoadjuvant therapy. However, these 5 cases did not show similarity in either regression rate or intrinsic subtype (Table 3).

**Table 3:** Comparison of ER staining in stromal cells in core biopsy before neoadjuvant treatment and after neoadjuvant treatment with regression rates

Before NAC/After NAC	(*n* = 299) (%)	Regression rate (%)	Pathologic complete response rate (%)
Negative/Negative	138 (46.1%)	68.4	23.2
Negative/Positive	132 (44.2%)	89.8	56.8
Positive/Positive	24 (8%)	88.1	54.1
Positive/Negative	5 (1.7)	79.3	40

*NAC* Neoadjuvant chemotherapy

## Discussion

The frequency of utilization of NAC for breast cancer is increasing day by day. Despite the existence of different models, so far, the pathologic assessment of tumor regression due to NAC for breast carcinoma has mainly been conducted by evaluation of the decrease in the neoplastic cellular volume. Estimating the decrease in the neoplastic cellular volume has been done by assessing the amount of reactive stroma and the distribution of residual neoplastic cells within the PCTB [1–6]. However, the biological characteristics of the tumor stroma may differ after the NAC, as it may happen in the neoplastic cells, and its determination may help in assessing the tumor regression and understanding how the tumor responds to chemotherapy.

It is well-known that chemotherapy can affect cancer cells and cells within the tumor stroma [15]. Myofibroblasts, especially CAFs, are also prominently found in the PCTB. The origin of CAFs is still debated, with theories ranging from mesenchymal cells derived from bone marrow to resident fibroblasts/myofibroblasts in the normal stroma and epithelial–mesenchymal transition [16–18]. A previous study suggested that resident fibroblasts in tumor tissues are the primary source of CAFs [19]. Kojima et al. engrafted healthy human breast fibroblasts and cancer cells into nude mice and found that the residential human breast fibroblasts were activated [20]. With this regard, ER positivity observed in stromal cells after the NAC may be suggested to be a supporter of the epithelial–mesenchymal transition theory that is considered secondarily effective during the chemotherapy.

Prior studies about chemotherapy response in breast carcinoma have mainly focused on ER expression in cancer cells without considering CAFs or stromal cells. Concerning ER expression in stromal cells, we have found only one study that has evaluated the relationship between ER and CAFs in gastric cancers [21]. Our study is the first to discover ER positivity in the stromal cells of the tumor bed in breast carcinoma patients who received NAC. The change in ER expression in the stromal cells (probably CAFs) after NAC observed in our study provides evidence for the significant effect of the treatment on the stromal cells. CAFs exhibit different gene expressions across various cancer subtypes. Tchou et al. reported significant differences in the gene expression of CAFs derived from HER2 + breast cancer, triple-negative breast cancer, and ER + breast cancer [22]. In our study, ER-positive stromal cells after chemotherapy were interestingly more frequent in HER2 overexpression type and triple-negative type invasive breast carcinoma cases as compared to luminal type. Therefore, in line with our results, it can be suggested that even in originally ER-negative invasive carcinoma cases, estrogen levels in the patient and ER status in the stromal cells may have a role in the degree of tumor response to chemotherapy.

Our study observed a statistically significant ER positivity in stromal cells along with well-response to NAC. We also found stromal ER positivity as an independent predictor for pCR. Briefly, the ER-positive stromal cells were more frequent in cases with well-response to NAC and are more frequent in non-luminal type breast carcinomas. In some cases, pre- and postchemotherapy specimens concerning ER expression in stromal cells were compared. In that manner, we demonstrated that original ER positivity in stromal cells was notably low in the prechemotherapy biopsies of these cases. Based on our findings, we may postulate that chemotherapy may induce a change in ER status in the stromal cells of the tumor, as it did in neoplastic cells in some breast carcinomas. It can be inferred that ER-positive stromal cells may augment the efficacy of chemotherapy on neoplastic cells in breast carcinoma. As another explanation for the appearance of ER-positive stromal cells after NAC, we suggest that they result from well-response to chemotherapy and a part of tissue organization after neoplastic cell apoptosis due to chemotherapy.

The weakness of our study is the utilization of only ER by using the immunohistochemical method. Nevertheless, our research is one of the pioneering studies. ER positivity in stromal cells has led to essential and practical findings regarding responses after NAC and may be helpful to other researchers studying neoadjuvant chemotherapy in breast carcinoma. Furthermore, some other markers may be used to define these stromal cells as CAFs, but to our knowledge, there is no specific marker for CAFs.

In conclusion, this is the first study regarding ER expression in the stromal cells of the PCTB. We showed that the increase in ER positivity in the stromal cells of the PCTB correlated with complete or well response. We also showed that a conversion to ER positivity in stromal cells was common in cases with well pathologic response after NAC. Considering that good chemotherapy response is one of the best prognostic indicators, ER positivity in the stromal cells of the tumor bed can be used as a new prognostic marker and predict the rate of chemotherapy response. After the treatment starts, showing stromal ER positivity in the core biopsy performed early in the treatment period can predict complete response. ER positivity in the stromal cells of the tumor bed will serve as a cornerstone in transforming the standard treatment option applied to all patients according to the protocol into a personalized treatment.

## Supplementary Information

Below is the link to the electronic supplementary material.

Supplementary file1 (DOCX 28 KB)

## Data Availability

No datasets were generated or analysed during the current study.
