# Supplementary material for: Estrogen receptor positivity in stromal cells of the tumor bed predicts the response to neoadjuvant chemotherapy for breast carcinoma
Source: Breast Cancer Res Treat. 2025 Jan 8;210(3):627–33. doi: 10.1007/s10549-024-07601-6 (PMC11953172; doi:10.1007/s10549-024-07601-6)
Supplement: Supplementary file 1 — Supplementary file1 (DOCX 28 KB) [file 10549_2024_7601_MOESM1_ESM.docx]

Supplement: Receiver operating characteristic (ROC) curve for ER staining in stromal cells in tumor bed


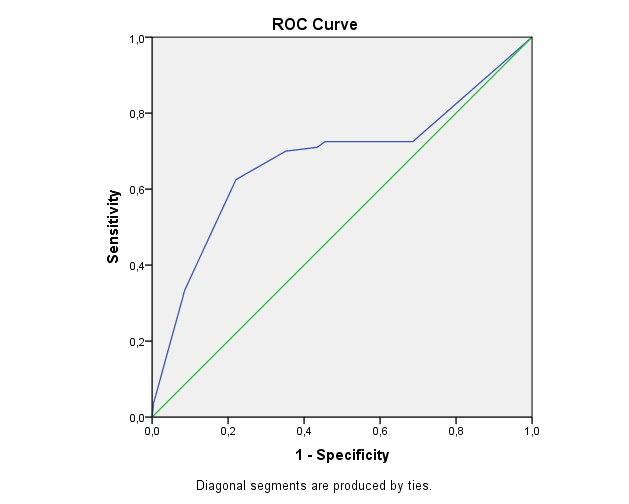


| **AUC (95% CI)** | **Cut-off (%)** | **Sensitivity (%)** | **Specificity (%)** | ***p*** |
| --- | --- | --- | --- | --- |
| 0,678 (0,626-0,730) | 55 | 70 | 64,8 | *<0,001* |

*AUC: Area under the curve, CI: Confidence Interval
